# Consumer Trust and Attitudes Towards Advertisements for Dietary Supplements

**DOI:** 10.3390/nu18152518

**Published:** 2026-08-03

**Authors:** Regina Ewa Wierzejska, Agnieszka Wiosetek-Reske, Anna Poznańska, Katarzyna Stoś

**Affiliations:** 1 Department of Nutrition and Nutritional Value of Food, National Institute of Public Health NIH—National Research Institute, Chocimska St. 24, 00-791 Warsaw, Poland; 2 Warsaw Medical and Technical Academy of Applied Sciences, 1920 Battle of Warsaw St. 18, 02-366 Warsaw, Poland; 3 Department of Population Health Monitoring and Analysis, National Institute of Public Health NIH—National Research Institute, Chocimska St. 24, 00-791 Warsaw, Poland

**Keywords:** dietary supplements, advertising, trust, knowledge, consumer

## Abstract

**Background:** The excessive use of dietary supplements is widespread, and their advertising is often misleading and suggests therapeutic properties. The aim of the study was to assess consumers’ perceptions of advertising, their expectations regarding the effects of dietary supplements, and their overall knowledge of these products. **Methods:** The study was conducted among 397 adults who were presented with the content of advertisements for four dietary supplements broadcast on Polish radio stations. **Results:** More than 67% of respondents had low or negligible knowledge of dietary supplements. Only 4.8% considered dietary supplements to be food products, whereas 40.3% believed them to be over-the-counter medicines. According to 45.7% of respondents, the advertisements created the impression that the efficacy of the products had been scientifically evaluated and proven. Furthermore, 74.1% believed that if the advertised dietary supplements did not have proven efficacy, such advertising was unfair and misleading. The largest proportion of respondents (41.1%) expressed moderate expectations regarding the efficacy of the advertised dietary supplements. Furthermore, the proportion of respondents expressing the highest level of scepticism increased with increasing knowledge of dietary supplements. Despite this, 60.6% of respondents declared that if they experienced the health problems referred to in the advertisement, they could be encouraged by it to start using such products. Factors that significantly increased the likelihood of dietary supplement use included high expectations regarding their efficacy (OR = 11.1), negligible knowledge about dietary supplements (OR = 2.9), the belief that their efficacy had been demonstrated in scientific studies (OR = 1.9), and residence in a rural area (OR = 1.9). **Conclusions:** Given forecasts of continued growth in the dietary supplement market, and the belief held by many consumers that the effects of such products have been proven, educational campaigns are needed to prevent them from being confused with medicines.

## 1. Introduction

Dietary supplements are now so widely used that concerns regarding their safety have become a matter of public health debate. The risks associated with taking supplements involve several factors, including excessive intake of nutrients or substances not normally found in the diet, adulteration with unauthorised substances, or a lack of standardisation regarding ingredient content [1,2,3,4,5,6,7]. The scientific literature unanimously points to a lack of public knowledge regarding the nature and properties of dietary supplements [8,9,10]. In Poland, in one of the most recent studies, as many as 43% of healthcare professionals surveyed (doctors, nurses, and pharmacists) stated that dietary supplements are medicines or medical devices, and nearly 50% believed that the legal requirements for the marketing of supplements are the same as those for medicinal products [11]. In the US, on the other hand, around 73% of the military physicians surveyed did not know where or how to report adverse reactions to these products [12].

Aggressive advertising, which presents dietary supplements as a simple solution to a wide range of health problems, plays a major role in their growing popularity [8,13,14,15,16]. According to a recently published survey of Polish pharmacy staff, as many as 90% of respondents expressed the view that the factor with the greatest influence on the purchase of these products by people over the age of 60 is advertising in the media [17]. Unfortunately, many people believe that dietary supplements have therapeutic effects, and experts are wondering why consumers place more trust in supplements that have not been tested in clinical trials than in medicines [14,18,19]. At the same time, the scientific literature frequently quotes the well-known saying: *“if a claim seems too good to be true, it probably is!”* [20,21,22]. Consequently, in order to raise consumer awareness, guidance documents aimed at the general public strongly emphasise the need to exercise particular caution, especially in the case of dietary supplements described as “magical”, “sensational” or representing a “scientific breakthrough” [21,23].

In Poland, as early as 2017, the Supreme Audit Office highlighted the need to monitor the advertising of dietary supplements due to unfair practices attributing therapeutic effects to these products, and experts in other countries have expressed the same view [4,14,15,24]. According to a ruling by the Court of Justice of the European Union, a product is presented as having properties capable of treating or preventing a disease not only when such a message is explicitly conveyed, but also if a reasonably well-informed consumer is led to believe this [25]. Similarly, the Japanese Consumer Affairs Agency states in its position paper that whether advertisements are misleading should be assessed on the basis of the impressions and perceptions that the message as a whole evokes in the general public, rather than on the basis of specific words or graphics [14]. In Poland, probably as a result of discussions regarding changes to the regulations governing the marketing of dietary supplements, television broadcasters and dietary supplement manufacturers established the “Broadcasting Agreement on the Rules of Advertising Dietary Supplements”, a self-regulatory framework that came into force on 1 January 2020 [26]. This agreement introduced a number of changes to the rules governing television advertising, including the requirement to display the following message on screen: *“Dietary supplement. Contains ingredients* that *support the body’s functions by supplementing the normal diet. Has no medicinal properties”*. However, according to a study by Wierzejska et al., which analysed advertisements for 46 dietary supplements following the agreement, nearly 30% of the advertisements still contained unauthorised claims suggesting that the product or its ingredients were effective against various health problems [13]. Legislative work is currently underway in Poland to increase the fines for non-compliance with requirements regarding the provision of information to consumers about food, including in the advertising and promotion of dietary supplements.

The use of dietary supplements is very widespread in Poland [27,28]. According to a survey carried out in 2022 across 14 EU countries, Poland has both the highest proportion of people reporting that they had taken supplements in the last 12 months (98% of respondents) and the highest number of supplements used (an average of 4 different products per year) [27]. At the same time, the pharmaceutical industry currently accounts for the largest share of the Polish advertising market. For example, over a four-week period (from 5 November to 2 December 2018) on the following television channels: Telewizja Polska, Polsat, and TVN; and on the following radio stations: Polskie Radio, Radio ZET, and RMF FM; the “medicines, dietary supplements and medical devices” category was the most frequently advertised product group, accounting for 28–38% of all television adverts and 23–43% of radio adverts. Within this category, dietary supplements accounted for 31% of the total, whilst the largest and smallest groups were medicines (53%) and medical devices (16%), respectively [29]. The literature, therefore, highlights the need to assess how purchasing behaviour is shaped by advertising for dietary supplements and consumers’ general understanding of these products [13,30,31].

There is almost no scientific data on consumers’ attitudes towards advertisements for dietary supplements. Given the huge number of advertisements and the legal requirements governing their content, it was therefore justified to find out how listeners perceive what they hear in radio adverts for dietary supplements, how they assess their reliability, and how the adverts influence their purchasing decisions. The decision to use radio adverts was based on the fact that, unlike television adverts, the reception of the message is not influenced by images on a screen, which may affect consumers’ overall impression.

## 2. Materials and Methods

### 2.1. Study Design

The study was conducted using a questionnaire containing the text of advertisements for four dietary supplements that were broadcast between 9 and 15 March 2020 on popular Polish radio stations (Polskie Radio Program I, Radio Zet, and RMF FM) and had previously been included in a pool of advertisements analysed by experts for compliance with the law [13]. The questionnaire was developed for the purposes of the study and had previously been tested on a group of around a dozen participants. Despite the use of hypothetical questions, such as ‘*would the advertisement encourage you*’ and ‘*if a product’s effects*’ understanding the questionnaire posed no problem, even for older people. On the day of the study, it was distributed to the participants by the researcher. The questionnaire was anonymous, and participants were informed that by completing it, they were giving their consent to participate in the study and that the results would be used solely for scientific purposes. Participants assessed adverts for products recommended for memory, weight loss, immunity, and sleep. The content of each advertisement was reproduced in the questionnaire in the form of text (as shown in Table 1). This cross-sectional study was carried out on a convenience sample of people living in medium-sized towns in central Poland. In total, the group comprised 397 adults aged 18 to over 65 years, of whom 62.7% were women, 57.2% had primary and secondary education, whilst 42.3% were students or had a higher education; 50.6% were urban residents and 48.6% were rural residents. The younger participants were secondary school pupils and university students, whilst the older participants were members of the Seniors’ Club and the University of the Third Age. The study included individuals who were present on that day and who were willing to complete the survey, and in the case of older people, those with good cognitive abilities.

A list of the questions included in the questionnaire, in English, is provided in the Supplementary File S1. The proportion of non-responses to individual questions, including those relating to personal details, did not exceed 0.8%. The respondents’ level of knowledge about dietary supplements was measured by the accuracy of their answers to six questions (Nos. 2–5, 14 and 15). Of the respondents, 4.5% did not provide complete answers—their level of knowledge could not be determined. Knowledge was classified as high for 4–6 correct answers, low for 2–3, and negligible for 0–1. The respondents’ expectations regarding the efficacy of the supplements were also assessed on the basis of their answers to six questions (Nos. 8, 9, 11, 13, 16 and 17). The percentage of missing data was 1.3%. If there were 5–6 affirmative answers, expectations were classified as high; if there were 3–4, as moderate; and if there were 0–2, as low. The authors believe that such assessment criteria fairly evaluate the parameters under study.

### 2.2. Statistical Analysis

Descriptive statistical methods were used to analyse the distributions of responses to individual questions in the questionnaire and the composite variables defined on the basis of those responses. The percentages reported in Section 3 relate to respondents who provided an answer to the respective question. The significance of differences in the frequency of responses given by groups of respondents categorised according to a categorical variable, including gender, level of education (primary or secondary vs. students or higher education), place of residence (urban vs. rural) or responses to other questions, was assessed using the chi-square test; for the three groups defined on the basis of ordinal variables (age, level of knowledge about supplements), the Cochran–Armitage trend test was used. This part of the analysis of the responses was exploratory.

Analysis of the consideration of the declared potential use of supplements resulting from advertising was conducted in two stages. The first was the identification of factors significantly related to the dependent variable. The univariate logistic regression method was used, and the corresponding odds ratios (OR), standard errors (SE), and 95% confidence intervals (95% CI) were calculated to assess the strength and statistical significance of the associations between individual factors and the dependent variable. To control for correlations between explanatory variables, the second step of the analysis was conducted using a multiple logistic regression model with explanatory variables (only categorical or ordinal) identified in the first stage. The discriminative performance of the resulting model was assessed using the AUC (*Area Under the Receiver Operating Characteristic (ROC) Curve*).

In all statistical tests, a significance level of 0.05 was assumed. The calculations were performed using Stata 16.1. and IBM SPSS Statistics 27.

## 3. Results

### 3.1. Respondents’ Knowledge of Dietary Supplements

Over 67% of respondents had low or negligible knowledge of dietary supplements (49.3% had low knowledge and 17.9% had negligible knowledge, respectively), whilst only 32.7% had a high level of knowledge. Although almost all respondents (97.2%) were familiar with the term “dietary supplement”, only 4.8% of them believed that dietary supplements were food, whereas as many as 40.3% stated that they were over-the-counter medicines and the remaining 54.8% believed them to constitute a separate category of products. Age and educational attainment were statistically significantly associated with the level of knowledge. The proportion of respondents with negligible knowledge decreased with age (from 20.8% among those aged 18–35 to 9.7% among those aged 36–60 and 4.6% among those aged over 61; *p* = 0.015), while students and respondents with higher education were significantly more likely to have a high level of knowledge than respondents with primary or secondary education (39.1% vs. 28.4%; *p* = 0.026). No difference was found in the level of knowledge about dietary supplements based on gender or place of residence.

When asked whether the statement “*contains natural ingredients*”, used in one of the advertisements, meant that the product was safer than one containing synthetically produced ingredients, respondents were evenly divided on this issue. Of the respondents, 50.1% and 49.4% answered “yes” and “no”, respectively. In turn, 30.5% of respondents believed that the word “*safe*”, used in another advertisement, meant that the product could be used without concern and did not pose any health risks. When asked whether the sale of dietary supplements in pharmacies indicates that they are subject to adequate control with regard to health standards and safety, 42.3% of respondents expressed this belief. The majority of respondents (88.4%) believed that one should consult a physician before taking dietary supplements for weight loss (this question was not included in the set of questions measuring general knowledge of supplements), and the proportion of such respondents increased with the level of knowledge about supplements (80.9% of respondents with negligible knowledge, 90.9% with low knowledge and 92.7% with high knowledge; *p* = 0.018).

Of the respondents, 60.0% answered the open-ended question “*What do you think about dietary supplements*?” Of these, 38% expressed a negative opinion, 35% a positive one, whilst the remaining 27% held a neutral view. The prevailing view amongst the negative opinions was that dietary supplements are not safe and do not inspire confidence. Among the positive opinions, respondents believed that dietary supplements were necessary for the body to function properly, and this view mainly concerned vitamin supplements. Among respondents expressing neutral opinions, the most frequently cited points were the need for a sensible approach, checking for nutrient deficiencies, and consulting a doctor.

### 3.2. Trust in Advertising

According to 45.7% of respondents, the content of at least one of the three adverts analysed in this respect gave the impression that the products’ efficacy had been assessed in scientific (clinical) trials involving human subjects and had been proven. This view was strongly correlated with knowledge of dietary supplements. People with low or negligible knowledge were statistically significantly more likely to be convinced that supplements were proven to work, compared with those with a high level of knowledge (53.9% vs. 28.2%; *p* < 0.001). No association was found between responses to this question and respondents’ age, gender, place of residence, or level of education in the analysed sample; however, it is worth noting that respondents with higher education were less likely to express this belief than the remaining respondents (31.3% vs. 47.7%; *p* = 0.032).

With regard to all three adverts analysed in this respect, 74.1% of respondents considered that if the advertised supplements do not have an effect proven by research, such advertising is unfair and misleading. Indeed, this view was expressed more frequently by people aged over 35 than by younger people (83.5% vs. 71.6%; *p* = 0.030) and by students and those with a higher education compared to those with primary and secondary education (79.5% vs. 69.8%; *p* = 0.030). This view was also held more frequently by women than by men (77.0% vs. 68.5%), but the difference observed was on the borderline of statistical significance (*p* = 0.066). One finding from the study worth highlighting is the consistency of views regarding the honesty of advertisements, regardless of respondents’ level of knowledge about dietary supplements. The percentage of respondents who stated that, in the absence of research confirming the efficacy of the products, the content of the adverts was dishonest differed by a maximum of 2.2% between the group with a high level of knowledge and those with a low or negligible level of knowledge. Views on the honesty of advertisements were also associated with having consulted a doctor about the use of weight-loss supplements. Among those who considered such advertising dishonest, 92.1% reported the need to consult a doctor, compared with 78.4% of those who did not question the honesty of advertisements in the absence of studies evaluating the effects of supplements (*p* < 0.001).

### 3.3. Expectations Regarding the Efficacy of Advertised Dietary Supplements

**Figure 1:**
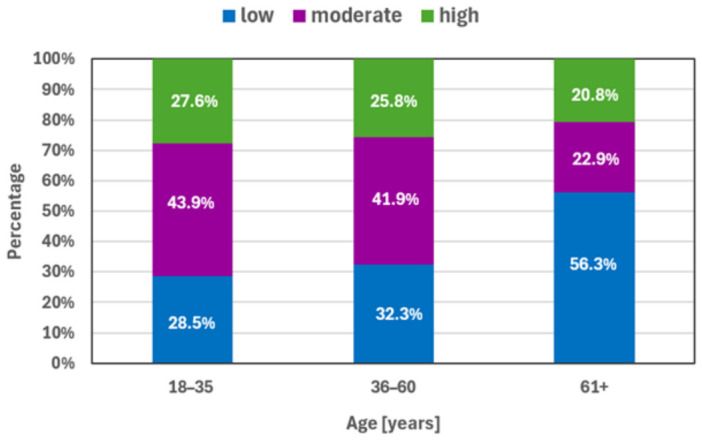
Expectations regarding the efficacy of advertised dietary supplements by respondents’ age.

The largest proportion of respondents (41.1%) expressed moderate expectations regarding the efficacy of the advertised supplements, 32.4% had low expectations, and 26.5% had high expectations. The level of expectations decreased significantly with respondents’ age (*p* = 0.005) (Figure 1).

In the case of a “memory” supplement, 57.7% of respondents stated that, if they experienced memory problems, they would expect noticeable improvements in memory based on the content of the advertisement, while 33.5% would expect the supplement to help maintain memory at an optimal level. According to 67.5% of respondents, this product should be used as a preventive measure to avoid memory decline, while 45.3% believed it was recommended for people who had already noticed memory problems. In the case of an advertisement for an “immunity-boosting” supplement, 73.1% of respondents believed that taking the supplement should result in an increase in the body’s immunity. Of the respondents, 60.2% stated that taking vitamins C and D and zinc in supplement form could boost immunity only in people who are deficient in these nutrients, whereas 30.2% believed this to be the case for everyone. Moreover, 59.7% believed that the more vitamins C, D and zinc we supply to the body, the better our immunity will be. When it comes to a “good night’s sleep” supplement, 69.3% of people expected it to have a calming effect, and 62.0% would expect it to be effective at helping them fall asleep. Respondents were most sceptical about “weight-loss” supplements, as only 31.3% believed that taking such a product would lead to weight loss.

**Figure 2:**
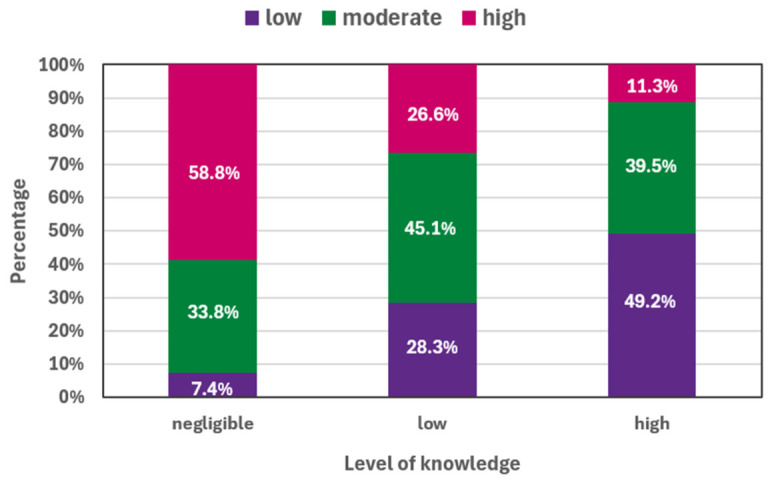
Expectations regarding the efficacy of advertised dietary supplements according to respondents’ knowledge of dietary supplements.

Furthermore, a strong association was found between expectations regarding the efficacy of products and the level of knowledge about dietary supplements (*p* < 0.001) (Figure 2). As knowledge increased, the proportion of respondents with high expectations regarding the effects of the advertised products decreased (from 58.8% of respondents with negligible knowledge to 11.3% of respondents with a high level of knowledge), while the proportion of respondents who were most sceptical about the efficacy of supplements increased from 7.4% to 49.2%.

The study also revealed a statistically significant association between the reported belief that the efficacy of supplements had been proven in scientific studies and the expected effects of these products. Among those who, based on the advertisement, believed that studies had proven the efficacy of the supplements, the proportion of people with high expectations was more than twice as high as the proportion of those with low or moderate expectations (37.4% vs. 17.0%; *p* < 0.001).

### 3.4. The Potential Use of Advertised Supplements

Of the respondents, with regard to at least one product, 60.6% stated that if they experienced the health problems mentioned in the advertisement, they might be encouraged by it to start using the advertised supplement. The proportion of such people varied in a statistically significant manner depending on age, level of knowledge and place of residence (Table 2). It was higher among rural residents than urban residents (67.0% vs. 54.5%; *p* = 0.011) and decreased markedly with age (from 63.9% amongst the youngest to 43.7% amongst the oldest respondents; *p* = 0.006) and as knowledge of dietary supplements increased (from 85.1% to 41.5%; *p* < 0.001). People who believed that the advertised products had been the subject of scientific research confirming their efficacy were also more likely to use them than those who were not convinced of this (74.3% vs. 48.8%; *p* < 0.001). Potential use was also strongly associated with respondents’ expectations regarding the efficacy of the supplements. If the health problems mentioned in the advertisement were to arise, 31.5% of respondents with low confidence in the efficacy of the products declared that they might use them, compared with 66.3% of those with moderate confidence and as many as 88.3% of those with high confidence that the advertised supplements work as presented in the advertisement (*p* < 0.001).

To eliminate the previously identified interdependencies between the explanatory variables, a multivariate logistic regression model was used. The model showed that the factors independently associated with the potential use of the advertised supplements were the level of knowledge about dietary supplements, the degree of belief in their efficacy, the belief that their efficacy had been confirmed in scientific studies, and place of residence (Table 3). A negligible level of knowledge increased the likelihood of using supplements by almost three times (OR = 2.85; *p* = 0.015), whilst a low level increased it by almost two times (OR = 1.72; *p* = 0.045) compared with a high level. Respondents with high expectations regarding the efficacy of supplements were 11 times more likely to use them (OR = 11.13; *p* < 0.001), while those with moderate expectations were more than three times more likely to do so (OR = 3.47; *p* < 0.001) than respondents with low expectations. People who were convinced that the efficacy of the advertised supplements had been proven in scientific studies were nearly twice as likely to use them if they encountered problems (OR = 1.93; *p* = 0.011) than those who were not convinced, whilst rural residents reported potential willingness to use such products almost twice as often as urban residents (OR = 1.89; *p* = 0.012).

**Figure 3:**
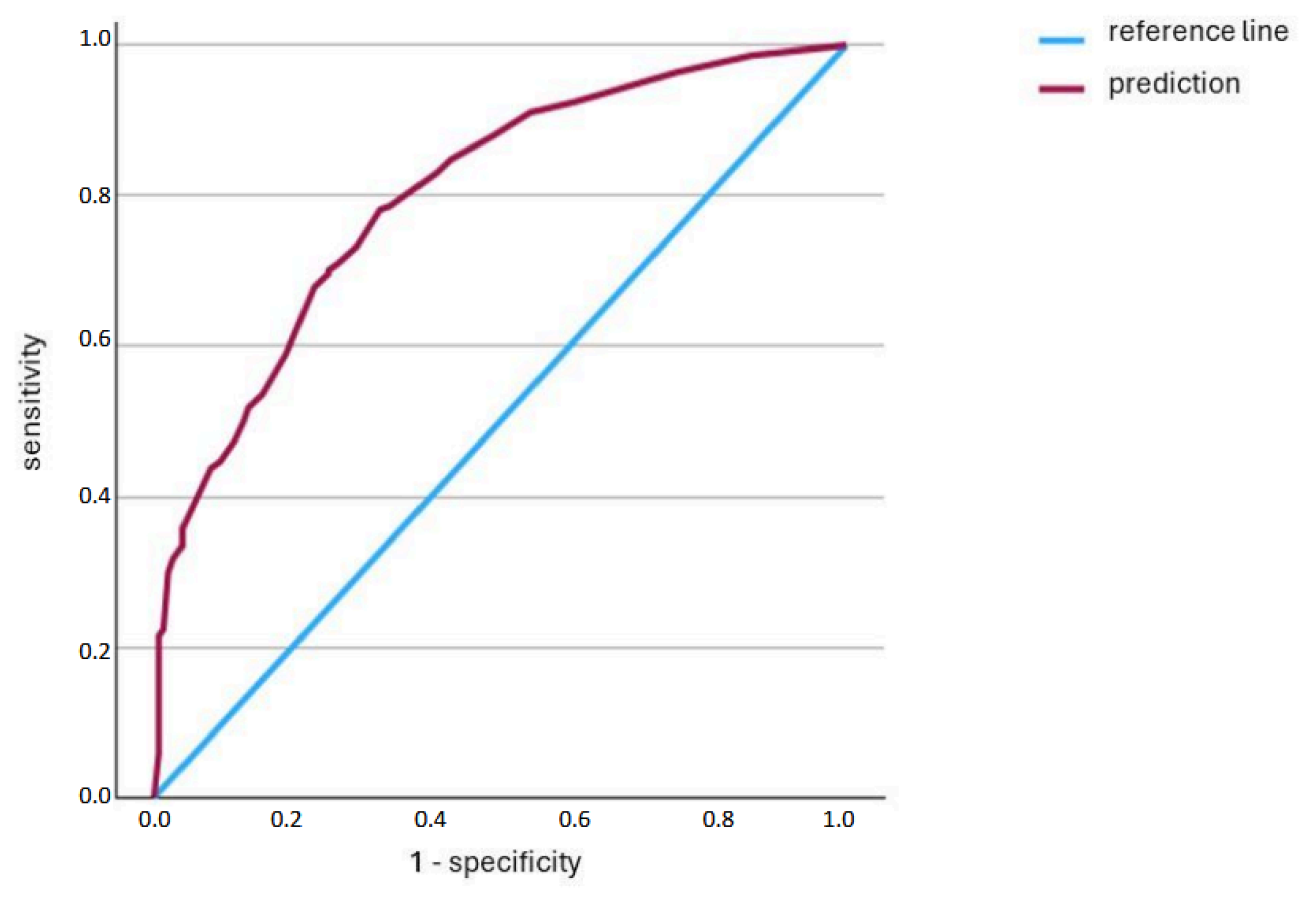
Receiver operating characteristic (ROC) curve for predicting the use of dietary supplements.

The resulting model proved to be a good predictor of the potential use of the advertised products (Figure 3). The Area Under the Curve (AUC), i.e., the area under the Receiver Operating Characteristic (ROC) curve, was 0.800 with a standard error of 0.023; the 95% confidence interval was 0.755–0.844.

## 4. Discussion

The basis of European Union food law is the protection of consumers against misleading information. Food information must not, among other things, claim that food products can prevent or treat diseases, or attribute properties to them that they do not possess. At the same time, the legislation clearly stipulates that all the above requirements apply equally to labelling and to advertising [32,33]. In 2006, the use of nutrition and health claims was regulated in detail [34], and in 2012 a list of authorised health claims that may be used when providing information about food was published [35]. However, there is a consensus in the scientific literature that advertisements for dietary supplements are misleading, often contain unauthorised health claims and are frequently accompanied by the catchy slogans “scientifically proven” or “guaranteed” [14,15,16]. Permitted nutrition claims, such as “*contributes to…*” and “*may help…*”, are often rephrased as assertive claims *such as* “*reduces…*”, “*increases…*” *and* “*improves…*” [15,36]. Consumers may therefore assume that a product available without a prescription, which can even be purchased in a grocery store, can be used for self-treatment of health problems.

There is little data on consumers’ perceptions of advertisements for dietary supplements. Among these, it is worth mentioning a Polish study by Karbownik et al., carried out on a group of 341 people aged between 18 and 77, which showed that trust in advertising is low. The median scores for reliability, comprehension, and emotional response ranged from 1 to 3 on a 7-point scale, where 1 represented the lowest score and 7 the highest [10]. In our survey, more than three-quarters of respondents stated that if there are no studies confirming that dietary supplements work as advertised, this is dishonest and misleading. Meanwhile, according to research to date, 30–44% of the radio and television advertisements analysed in Poland contained unsubstantiated claims regarding the efficacy of dietary supplements in various health-related situations [13,36]. It is currently believed that false or misleading information about supplements is further fuelled by influencer marketing on social media. According to data from the Media Monitoring Institute, in June 2022, there were around 3600 posts on Polish social media about dietary supplements, of which only 3% were from specialists [37]. In an American study, as many as 97% of the TikTok videos analysed relating to dietary supplements contained claims about health benefits, without any scientific evidence to support them [38], whilst on Instagram, a single post contained as many as 21 claims, including, among others, claims about preventing glaucoma and Alzheimer’s disease [39]. According to advertising experts, listing numerous features and benefits of a product is intended to overwhelm the audience with information and create the impression that the product in question must be highly effective [40].

As regards the expected efficacy of advertised supplements, the majority of respondents in our study expressed moderate expectations, indicating a relatively sensible consumer approach. Moreover, expectations regarding supplement efficacy decreased as respondents’ knowledge increased, as has also been observed in other studies [10,31]. However, the expected efficacy did not directly translate into a declaration regarding the potential use of supplements to address the problems highlighted in the advertisement. Unfortunately, this study adds to the body of evidence suggesting that the level of knowledge about dietary supplements is low [8,10,11,41,42]. An exceptionally low proportion of respondents (just under 5%) correctly classified these products as a type of food, which is the lowest result compared with other Polish studies (44–65%) [8,10,11]. However, a lack of understanding of what dietary supplements are is not unique to Polish society; in Germany, for example, only 14% of consumers regarded dietary supplements as food [43]. Nevertheless, the proportion of respondents who incorrectly classified dietary supplements as over-the-counter medicines was comparable to that reported in other Polish studies and, unfortunately, remains high (40% vs. 41–43%). This misconception is also widespread in Poland among medical professionals [8,11]. The study also confirms that a large proportion of respondents are quick to assume that supplements containing natural ingredients are safer, and that for many people, the use of the word “safe” in advertising implies that such a supplement can be taken without any health concerns. The view held by consumers that, since dietary supplements are classified as food, they must be safe, or that their safety stems from the fact that they are produced from natural ingredients or herbs, has been noted on numerous occasions [6,44,45].

The level of knowledge about dietary supplements also proved to be one of the factors that may influence the use of advertised supplements. This correlation had already been demonstrated in an earlier Polish study, in which a higher level of knowledge was negatively correlated with the use of supplements [10]. The result we obtained also seems reasonable: a greater belief in the efficacy of products encourages their use. Indeed, people who take dietary supplements are more likely to believe that these products can prevent or treat diseases than those who do not take supplements [44]. At this point, it is important to support the call—which has been made repeatedly—for educational campaigns to be conducted, in response to the increasing advertising of dietary supplements, aimed at both healthcare professionals and the general public [10,46]. It is worth quoting here the very apt title of an American campaign on dietary supplements—“Supplement Your Knowledge” [46].

The sale of dietary supplements in pharmacies—where these products are most commonly purchased [8,47,48]—also reinforces the perception that they are safe, as other authors have previously demonstrated [10]. It is well known, however, that dietary supplements are not subject to safety testing, and, as some claim, they are only tested by the people who buy them [49]. Despite this, in Norway as many as 96% of patients are convinced that dietary supplements undergo safety testing before being placed on the market [42], whereas in Poland this proportion is approximately half as high, although it still accounts for almost half of respondents (43–46%) [10]. Across the European Union, as many as 72% of consumers surveyed said they were confident in the safety and quality of these products [27].

The public also places high expectations on vitamin supplementation. In our study, nearly 60% of respondents believed that the greater the intake of vitamins C and D and zinc, the better immunity would be, whilst in another Polish study, as many as 80% of respondents believed that taking vitamin C regularly reduces the risk of catching a cold [10]. Such beliefs are also common in other countries [42]. Although pharmacists are the people from whom reliable advice on dietary supplements is expected [50,51], it must not be forgotten that pharmacies are businesses that need to make a profit from their operations. In this context, as many as 85% of staff at Polish pharmacies believe that dietary supplements are an important source of income [17].

Despite the fact that the proportion of respondents stating that, should the problems mentioned in the advertisement arise, they might start taking the advertised supplements (around 60%) was higher than that reported in other Polish studies, this comes as no surprise, given the recent popularity of dietary supplements. However, it should be remembered that the reported willingness to use dietary supplements after exposure to advertising may not translate into real purchasing decisions. In a previous study by Wierzejska et al., almost 37% of patients stated that they considered advertising when buying a dietary supplement [8], whilst in a study by Strocka et al., over 20% of Poles surveyed replied that their choice of dietary supplement was based on advertising [52]. A slightly higher proportion was found among healthcare professionals, with more than 30% agreeing that advertising plays a role in the choice of supplements [11]. Meanwhile, according to 84% of the pharmacists and pharmacy technicians surveyed in Poland, the main measure to curb the misuse of dietary supplements by older people would be a ban on the advertising of these products in the mass media [17].

When discussing the results of the study, its limitations should be highlighted. The main one is that it was theoretical in nature. For this reason, participants’ responses may differ from those that would be obtained under real radio advertising conditions, where different speaking rates, intonation and voice volume are used. On the other hand, a radio listener’s attention may be distracted by whatever they are doing at that moment, which may affect the selectivity of the information they pick up. Such factors were not present in the conditions of our study, where participants were presented with the advertisement text and were able to read it carefully, and even several times. All of this can influence how advertisements are received and, consequently, how the advertised products are perceived. It is therefore not certain that the participants’ statements in a study conducted in this way would correspond to their perception of the advertisements and their behaviour in real-life situations. Furthermore, this was a cross-sectional study conducted on a convenience sample of participants and therefore no general conclusions should be drawn.

To the best of the authors’ knowledge, however, this is the first study in which respondents were shown specific advertisements for dietary supplements with a view to analysing how they were received. Only four dietary supplements were included, which, given the size of the market and the scale of advertising for such products, may be considered symbolic. Nevertheless, respondents’ perceptions and the amount of time they were able to devote to completing the questionnaire should be taken into account. It is also clear that the way advertisements are perceived is highly individual, and there are probably no advertisements that are perceived in exactly the same way by all recipients [40]. It is also impossible for the general public to analyse advertisements and draw their own conclusions; therefore, this type of pilot study conducted by researchers makes a valuable contribution to the ongoing debate on the need to amend the legislation governing dietary supplements.

## 5. Conclusions

The content of advertisements for dietary supplements may lead many people to believe, mistakenly, that the effects of these products on the body, as described in the advertisements, have been confirmed by scientific research. At the same time, the majority of respondents believe that, if no such research has been carried out, advertisements designed in this way are unfair and misleading. However, despite having moderate expectations regarding the efficacy of the supplements, most respondents would be willing to start taking them if they experienced health problems. Nevertheless, it should be borne in mind that the study was theoretical in nature, and the participants’ declarations may not necessarily correspond to their behavior in everyday life. Given the forecasts for the continued growth of the dietary supplements market and the prevalence of misleading advertising practices, educational campaigns are needed to raise awareness of the nature of these products, in order to prevent them from being confused with medicines.

## Figures and Tables

**Table 1:** Content of the dietary supplement advertisements analysed.

Type of Dietary Supplement	Text of the Radio Advertisement
For memory	*“I teach history at school. In my job, knowledge and a good memory are essential. There are so many things I have to remember—important dates, events, facts, and the names of rulers and kings. That’s why I use (product name) to keep my memory in top condition. The ingredients in the dietary supplement (product name) help to maintain concentration and mental alertness. “(Product name) and I remember everything.”*
For weight loss	*(Product name)—a dietary supplement for weight loss. Tried and tested, safe, effective.*
For immunity	*I use (product name). Why? (Product name) supports the immune system. (Product name) is a dietary supplement containing the maximum permitted amount of vitamin C in a sustained-release form. And on top of that, it’s rich in zinc and vitamin D. This combination is a powerhouse for the immune system.*
For sleep	*Can’t get to sleep? (Product name) means a good night’s sleep for me. Contains natural ingredients. I’m taking (product name)—there’s no point in waiting. Dietary supplement (product name): A healthy dose of sleep. Hop cone extract helps promote restful sleep, whilst extract of saffron crocus helps promote emotional balance. Your racing thoughts no longer prevent you from falling asleep.*

**Table 2:** Percentage of respondents declaring potential use of the advertised supplements, by factors associated with the frequency of this declaration.

Factor	Number of Respondents Included in the Analysis, (%)	Percentage of Respondents Declaring that they Would Use Supplements if Needed, (%)
Age [in years]		
18–35	313 (79.8%)	63.9%
36–60	31 (7.9%)	54.8%
>61	48 (12.2%)	43.7%
Respondents’ level of knowledge		
high	123 (32.7%)	41.5%
low	187 (49.3%)	63.6%
negligible	67 (17.9%)	85.1%
Place of residence		
urban	201 (50.6%)	54.5%
rural	193 (48.6%)	67.0%
The belief that scientific research has confirmed the efficacy of the advertised supplements		
yes	179 (45.7%)	74.3%
no	213 (54.3%)	48.8%
Expectations regarding the efficacy of advertised supplements		
low	127 (32.6%)	31.5%
moderate	160 (41.0%)	66.3%
high	103 (26.4%)	88.3%

**Table 3:** Factors associated with the potential use of advertised dietary supplements.

Factor	Odds Ratio (OR)	Standard Error OR–SE (OR)	95% Confidence Interval (95% CI)	Statistical Significance (*p*-Value)
Level of knowledge about dietary supplements:				
- negligible *	2.85	1.23	1.23–6.62	0.015
- low *	1.72	0.46	1.01–2.92	0.045
Expectations regarding the efficacy of advertised supplements:				
- moderate level **	3.47	0.96	2.03–5.95	<0.001
- high level **	11.13	4.36	5.16–23.99	<0.001
The belief that the efficacy of supplements has been proven in scientific studies	1.93	0.50	1.16–3.20	0.011
Rural place of residence	1.89	0.47	1.15–3.08	0.012

* compared with the high level. ** compared with the low level.

## Data Availability

The original contributions presented in this study are included in the article/Supplementary Materials. Further inquiries can be directed to the corresponding author.

## Supplementary Materials

The following supporting information can be downloaded at: https://www.mdpi.com/article/10.3390/nu18152518/s1, Supplementary File S1: Questions asked to survey participants.
