# Supplementary material for: Consumer Trust and Attitudes Towards Advertisements for Dietary Supplements
Source: Nutrients. 2026 Aug 3;18(15):2518. doi: 10.3390/nu18152518 (PMC13468536; doi:10.3390/nu18152518)
Supplement: Supplementary file 1 [file nutrients-18-02518-s001.zip › nutrients-4436312-supplementary.pdf]

Supplementary File S1

Questions asked to survey participants

1. Have you ever come across the term "dietary supplement"?
2. In your opinion, are dietary supplements:
   - food
   - over-the-counter medicines
   - another product group
3. Does the fact that dietary supplements are sold in pharmacies mean that they are subject to sufficient quality and safety controls with respect to human health?
4. Does the phrase "contains natural ingredients" in the advertisement mean that the product is safer than one containing synthetically produced ingredients?
5. Does the word "safe" in the advertisement mean that the product can be used without concern and does not pose any health risks?
6. Does the content of the advertisement give the impression that the supplement's effects have been evaluated in scientific (clinical) studies involving human participants and scientifically proven? (This question was asked three times, in relation to advertisements for supplements "for memory", "for weight loss", and "for sleep".)
7. Do you think that if a product's effects have not been proven in scientific studies, such an advertisement is unfair and misleading? (This question was asked three times, in relation to advertisements for supplements "for memory", "for weight loss", and "for sleep".)
8. Based on the content of the advertisement, do you believe that using this product helps maintain memory at an optimal level?
9. If you were experiencing memory problems, would you expect this product to produce noticeable improvements in your memory?
10. Based on the content of the advertisement, for whom do you think this product is recommended? (you may select more than one answer.)
    - for people with memory problems
    - as a preventative measure to avoid memory decline
11. Based on the content of this advertisement, do you expect that the product causes weight loss?
12. Should a doctor be consulted before taking dietary supplements for weight loss?
13. Based on the content of this advertisement, do you think that taking the product should result in an increase in the body's immunity?

14. Do you think that the more vitamin C, D and zinc the body receives, the better its immunity will be?
15. Do you think that taking vitamins and minerals in supplement form may support the immune system (select one answer.)
    - in all people
    - only in those who have a deficiency of these nutrients
16. Based on the content of this advertisement, do you expect the product to have a sleep-inducing effect
17. Based on the content of this advertisement, do you expect the product to have a calming effect
18. If you experienced memory problems, would the content of the advertisement encourage you to use this product?
19. If you experienced overweight or obesity-related problems, would the content of the advertisement encourage you to use this product?
20. If you experienced sleep problems, would the advertisement encourage you to start using this product?
21. What is your opinion on dietary supplements ……………………………….
